# Signaling at Physical Barriers during Pollen–Pistil Interactions

**DOI:** 10.3390/ijms222212230

**Published:** 2021-11-12

**Authors:** Kayleigh J. Robichaux, Ian S. Wallace

**Affiliations:** 1 Department of Biochemistry and Molecular Biology, University of Nevada, Reno, NV 89557, USA; krobichaux@nevada.unr.edu; 2 Department of Chemistry, University of Nevada, Reno, NV 89557, USA

**Keywords:** pollen penetration, plant cell wall, pectin, cell–cell interaction, cell adhesion

## Abstract

In angiosperms, double fertilization requires pollen tubes to transport non-motile sperm to distant egg cells housed in a specialized female structure known as the pistil, mediating the ultimate fusion between male and female gametes. During this journey, the pollen tube encounters numerous physical barriers that must be mechanically circumvented, including the penetration of the stigmatic papillae, style, transmitting tract, and synergid cells as well as the ultimate fusion of sperm cells to the egg or central cell. Additionally, the pollen tube must maintain structural integrity in these compact environments, while responding to positional guidance cues that lead the pollen tube to its destination. Here, we discuss the nature of these physical barriers as well as efforts to genetically and cellularly identify the factors that allow pollen tubes to successfully, specifically, and quickly circumnavigate them.

## 1. Introduction

In angiosperms, the productive interaction between male and female gametes resulting in the formation of a new embryo and surrounding endosperm tissue is known as double fertilization. In contrast to many animal systems, sperm cells in higher plants are non-motile and thus rely on a specialized structure associated with the male gametophyte, known as the pollen tube, to move sperm cells to the egg. Additionally, the female reproductive tissue surrounding the egg cell is structurally complex and inaccessible to the male gamete. Therefore, the pollen tube must invade these tissues, circumvent numerous physical barriers, and discharge sperm cells to complete the fertilization process. Communication between pollen and pistil tissues is essential to mediate these successful fertilization events.

The pollen grain initially interacts with specialized finger-like cells known as stigmatic papillae, and if this interaction is productive, the pollen grain will be hydrated by the stigmatic papillae cells and subsequently germinate to form a pollen tube [1]. The pollen tube penetrates through the stigmatic papillar cell and then into the style tissue before entering a tube known as the transmitting tract (TT). Once in the TT, the pollen tube must escape this structure, migrate along the funiculus, locate an unfertilized ovule, and penetrate through the filiform apparatus and specialized synergid cells to gain access to the egg cell. After synergid penetration, the pollen tube bursts and releases two sperm nuclei that fuse with the egg and central cells to form a new embryo and new surrounding endosperm tissue, respectively.

**Figure 1:**
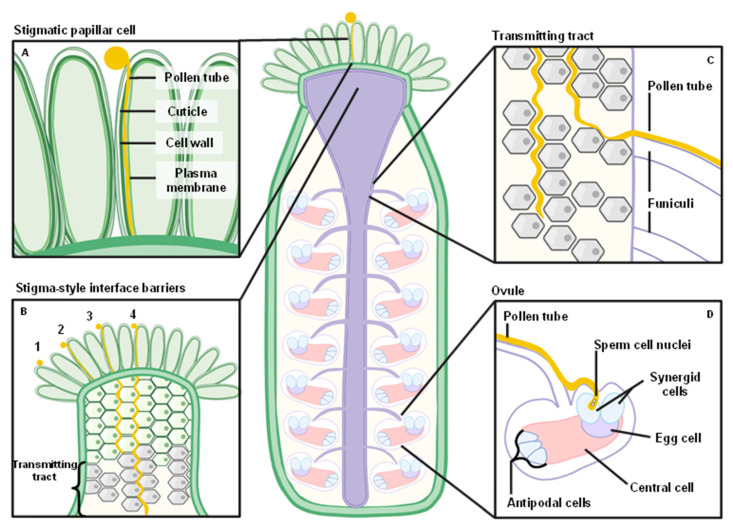
Schematics of physical barriers encountered by the pollen tube. An Arabidopsis pistil is shown with various physical barriers that are encountered by the pollen tube. (**A**) Pollen grain foot formation, hydration and subsequent pollen tube germination occurs at the stigmatic papillae, and pollen tube growth occurs between the cell wall and plasma membrane layers. (**B**) Possible barriers encountered by the pollen tube are displayed including penetration of (1) the stigmatic papillae, (2) the stigma-style interface, and (3) the transmitting tract. The fourth pollen tube has successfully penetrated through these barriers and is descending through the transmitting tract. (**C**) Germinating pollen tubes navigating the transmitting tract receive guidance cues that prompt pollen tubes to exit the transmitting tract tissues and begin moving along a funiculus to reach the ovule. (**D**) The ovule is penetrated by the pollen tube via the micropylar opening. The pollen tube then penetrates the degenerating synergid cell. Subsequent bursting and delivery of the sperm cell nuclei are imminent.

Along the pollen tube’s trajectory, the female tissue imposes numerous physical barriers (Figure 1), including (i) penetration of the pollen tube into the stigmatic papillar cell, (ii) penetration through the style and TT, (iii) exit of the pollen tube from the TT, and (iv) penetration of the pollen tube into the ovule for rupture and sperm discharge. For the pollen tube to successfully navigate each of these physical barriers, complex signaling and cell–cell communication events are required [1,2,3]. Here, we will discuss the facets of pollen tube growth that facilitate this amazing journey, the response of pollen tubes to both inanimate and physiologically relevant physical barriers, and the signaling processes that are known to occur at each of these migratory waypoints, with a primary focus on *Brassicaceae* species with dry-type stigmas.

## 2. Pollen Tube Growth and Cell Wall Composition

Pollen tubes grow both in vitro and in vivo via a specialized process called tip growth in which the tip of the pollen tube elongates in a polarized fashion [4,5,6]. Pollen tubes are the fastest growing cells in land plants, and thus, it is important to consider the factors that facilitate this robust growth. Internally-generated turgor pressure drives pollen tube tip expansion, and this force is counteracted by the pollen tube cell wall. Additionally, the pollen tube cell wall is likely a primary interaction site between the pollen tube and invaded cells within pistil tissue. Therefore, changes in pollen tube or pistil cell wall chemistry have the potential to modulate cell–cell adhesion and cell–cell communication during the process of double fertilization.

Plant cell walls are complex polysaccharide-rich extracellular matrices that commonly consist of three functionally distinct but interconnected polysaccharide networks: cellulose, neutral hemicelluloses, and acidic pectins [7,8]. Pollen tube cell walls contain all of these common polysaccharide networks [9], but compared to somatic cell walls, pollen tube cell walls exhibit some striking structural differences, including a lower relative abundance of the paracrystalline β-(1→4)-linked glucan cellulose [9], substantially increased abundance of the β-(1→3)-linked glucan polymer callose, and an ordered pattern of pectic polysaccharide deposition. These differences in cell wall composition are thought to confer pollen tubes with additional stability while they rapidly grow. The callose layer is considered a secondary wall that supports the pectin-cellulose complex [10,11], resisting the substantial turgor pressure required for pollen tube tip growth. Pectin is a major polysaccharide component of pollen tubes that consists of homogalacturonan (HG), rhamnogalacturonan-I (RG-I), and rhamnogalacturonan-II (RG-II). HG is composed of α-(1→4)-linked *D*-galacturonic acid residues that can be modified with acetyl or methyl esters [9]. Methyl esterification is particularly important in pollen tube HG structure because contiguous segments of de-methyl esterified galacturonic acid residues can be crosslinked via calcium ions to form more compact “egg box” structures that lead to increased cell wall rigidity. Flowering plants contain highly methyl esterified HG at the tip of the pollen tube, and weakly methyl esterified HG in the pollen tube shank [9,12]. This gradient of HG modification is postulated to increase the cell wall rigidity at the shank, while leaving the tip of the pollen tube flexible enough to expand [9,10].

HG synthesis requires the activity of homogalacturonan:galacturonosyltransferases (GAUTs) [13,14]. In Arabidopsis, two of the 15 *GAUT* genes (*GAUT13* and *GAUT14*) are highly expressed in pollen tubes. Analysis of *gaut13* and *gaut14* loss-of-function single mutants did not reveal a substantial impact on pollen tube function, but double *gaut13*/*gaut14* mutants produced pollen tubes that germinate but fail to elongate and additionally exhibited tip rupture under both in vitro and in vivo conditions [15]. These experiments also revealed that *gaut13*/*gaut14* double mutants are devoid of de-methyl esterified HG in the pollen tube shank, but retain methyl esterified HG at the pollen tube tip. These observations indicate that *GAUT13* and *GAUT14* redundantly participate in the synthesis of pollen tube HG, and further indicate that this HG deposition is required for pollen tube elongation.

RG-I and RG-II biosynthesis also appear to be critical for pollen tube elongation, although the biosynthesis of these polysaccharides is relatively poorly understood. RG-I is structurally composed of a backbone that consists of a repeating rhamnose α-(1→4)-galacturonic acid α-(1→2)-disaccharide [16]. This backbone structure can be further elaborated by complex arabinan, galactan, and arabinogalactan side chains [17]. Despite the fact that very few enzymes implicated in pectic RG-I synthesis have been identified, prior work shows that pollen tubes contain RG-I-associated arabinan epitopes [11], indicating that RG-I is present in the pollen tube cell wall. Additionally, a DUF-246 family glycosyltransferase of unknown function called PECTIC ARABINOGALACTAN SYNTHESIS-RELATED (PAGR) was recently described to play a role in RG-I arabinan side chain biosynthesis in Arabidopsis, and *pagr* mutants produced defective pollen tubes that were unable to elongate under in vitro conditions and did not transmit in self-fertilization assays [18]. These observations suggest that RG-I deposition is critical for proper pollen tube function.

RG-II is an exceptionally complex polysaccharide that exhibits structural conservation across the green lineage [16]. This polysaccharide consists of an α-(1→4)-linked galacturonic acid backbone with four structurally conserved side chains containing 12 different monosaccharides in 21 different glycosidic linkages [17,19]. Monomers of this polysaccharide are capable of self-dimerization by the formation of boric acid-mediated cross-links attached to apiose residues within RG-II monomers, and it is postulated that these cross-links further contribute to cell wall rigidity. Although the biosynthesis of RG-II is poorly understood, several pieces of evidence indicate that RG-II biosynthesis is essential for pollen tube elongation. For example, boron deficiency severely impacts pollen development [20,21], suggesting that boric acid crosslinking of RG-II is critical for these processes. Additionally, several postulated RG-II biosynthetic genes have been identified, but in almost all cases, mutations in these genes have resulted in defective pollen tube elongation and gametophytic lethality [22,23,24,25,26], which has precluded their further analysis. Thus, these observations indicate that RG-II is essential for pollen tube elongation in vitro and likely in vivo when penetrating through the female reproductive apparatus.

## 3. Pollen Tube Behavior during Encounters with Inanimate Barriers

Investigating pollen tube growth through the pistil tissue would be an ideal way to study physical barriers associated with this process. Although various methodologies are used to realize this goal [27,28], this scenario imposes several critical limitations, including (1) asynchronous germination of pollen tubes, (2) crowding of pollen tubes within the pistil, (3) lack of control on the complex pistil biological system, and (4) the requirement for advanced imaging methods to visualize pollen tubes in living pistils. To circumvent some of these challenges, microfluidic platforms have been exploited to provide defined microenvironments for single-cell analysis of pollen tubes and address key biophysical questions about their behavior during the process of pistil penetration.

TipChip [29], a microfluidic Lab-On-Chip (LOC) device, was developed to evaluate pollen tube behavior during elongation through a series of increasingly narrow gaps that mimic the conceivably narrow openings in the pistil that pollen tubes encounter. TipChip negates the effect of pollen tube-secreted hydrolytic enzymes that could potentially confound the biomechanical properties of the pistil tissue and allows for the isolated examination of a pollen tube’s ability to circumvent narrow openings of controlled size. *Camellia japonica* pollen tubes with a diameter ranging from 13–21 µm were germinated and directed to navigate a series of microgaps ranging from 17 to 10 µm. The pollen tubes were imaged during their growth trajectories, and several outcomes were observed, including successful navigation of the narrow opening, pollen tube bursting, or stalling. The frequency of these outcomes was linked to the ratio of opening size versus pollen tube diameter with smaller microgap sizes being associated with bursting or stalling. Similar work on *Torenia fournieri* pollen tubes resulted in similar conclusions [30], demonstrating that pollen tubes could navigate microgaps as small as 1 µm and further illustrating that sperm cells are capable of navigating these barriers within the compressed pollen tube. These findings suggest that pollen tubes behave similar to balloon catheters that are conventionally used to widen blocked arteries by significantly deforming temporarily while invading through very small spaces. However, these studies also highlight the fact that there are limits to the degree to which pollen tubes can maintain structural integrity while deforming, which is quite important due to the fact that cell–cell junctions in somatic tissues such as the pistil are estimated to be approximately 100 nm in width. Overall, these experiments suggest that pollen tubes are capable of drastically deforming to navigate small openings that are slightly smaller than the width of the pollen tube, but also suggest that additional factors, such as targeted cell wall deconstruction, may be necessary for the pollen tube to navigate the compact gaps that are experienced in the pistil.

## 4. Physical Interactions with the Stigmatic Papillae

One of the first barriers encountered by the pollen grain and subsequently formed pollen tube are the stigmatic papillae cells (Figure 1A). The pollen grain is deposited atop the stigmatic papillar cell, and this interaction results in the formation of an adhesive structure known as the pollen “foot” [31]. If the pollen grain is compatible, the papillar cell hydrates the pollen, resulting in the formation of a pollen tube that penetrates into the papillar cell by growing through the cuticle and subsequently between layers of the cell wall [32,33,34,35,36]. The process of entering the stigmatic cuticle would plausibly require enzymatic digestion and deconstruction of these layers by cutinases or cell wall degrading enzymes to facilitate pollen tube entry.

While the identity of plant cutinases has remained somewhat enigmatic, an Arabidopsis GDSL family esterase known as CUTIN DESTRUCTING FACTOR1 (CDEF1) caused substantial cutin degradation in transgenic plants over-expressing this enzyme [37]. Intriguingly, *CDEF1* transcripts are highly expressed in pollen during initial interactions with the stigmatic papillae, but *cdef1* null mutant pollen are capable of normal fertilization, suggesting that additional cutin degrading factors may be important for pollen tube entry through the cuticle.

Once the pollen tube circumvents the papillar cell cuticle, it must rapidly elongate through the space between layers of primary and secondary cell wall [35]. Although a substantial body of work has been devoted to elucidating the initial signaling events that impact pollen tube hydration and germination, the mechanistic basis of this penetration step remains quite enigmatic. Recently, an elegant live-cell imaging and electron microscopy analysis revealed that cellulose microfibrils and their oriented deposition, controlled by cortical microtubules (CMTs) [38], may play an important role in guiding this growth process [35]. Here, the authors demonstrated that CMTs progressively lose their anisotropic orientation perpendicular to the papillar cell growth axis during normal development, in *KATANIN* mutants, or in response to the microtubule depolymerizing drug oryzalin. The loss of CMT anisotropy generally led to loss of cellulose microfibril anisotropy and caused pollen tube coiling around the papillar cell, suggesting that cellulose microfibrils deposited in an anisotropic orientation contribute to pollen tube guidance through the papillar cell wall. The authors further demonstrated by Atomic Force Microscopy (AFM) that *ktn1-5* loss-of-function mutants had cell walls that were softer than wild-type papillar cells at the same developmental stage, indicating that wall stiffness surprisingly plays a role in efficient pollen tube growth through the papillar cell. These observations highlight the importance of both the cuticle and cell wall in pollen tube invasion at the papillar cell, but also indicate that the processes that actuate this invasive behavior are largely unclear.

## 5. Penetrating through the Stigma-Style Interface

**Figure 2:**
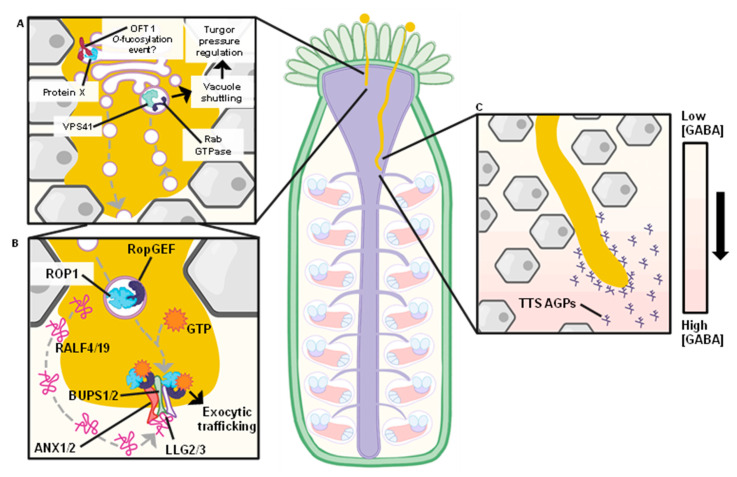
Schematic of physical barriers encountered by a germinating pollen tube at the style and transmitting tract. (**A**) Enlarged view of a germinating pollen tube navigating the tightly-packed style tissue. (**B**) The ANX1/2-BUPS1/2-LLG2/3 signaling complex is localized to the tip of the growing pollen tube and binds pollen tube-secreted RALF4/19, functioning as an autocrine signaling loop to prevent premature bursting. RopGEFs interact with the ANX1/2-BUPS1/2-LLG2/3 complex while activating ROP1, resulting in exocytic trafficking that may be essential for mechanotransduction signaling to occur for the pollen tube to exit the stigma-style interface (**C**) As the pollen tube penetrates further down the loosely packed transmitting tract, the pistil-produed γ-Amino-Butyric Acid (GABA) concentration gradient grows higher and Transmitting Tissue Specific (TTS) Arabinogalactan Proteins (AGPs) increase in abundance, both signaling to the pollen tube that it is navigating in the correct direction.

After penetrating through a papillar cell, the pollen tube must begin to elongate through the apoplastic spaces of tightly associated cells within the style (Figure 1B and Figure 2). During this time, numerous transcriptional changes occur in the pollen tube that prepare it for subsequent phases of the fertilization process [39,40,41]. For example, a genome-wide transcriptomic analysis has revealed that pollen tubes grown in vitro have a significantly different transcriptome compared to pollen tubes grown under semi-in vivo (SIV) conditions in which they have penetrated the stigma-style interface. This observation suggests that a novel set of transcripts are induced while the pollen tube grows inside the pistil tissue [39,42], and this hypothesis is consistent with pollen tube phenotypic changes during pistil interaction, such as significantly faster extension times [39]. The SIV pollen tube transcriptome contained approximately 380 genes that were unique to this experimental condition, suggesting that these genes are responsible for pollen tube capacitation during growth through the pistil. TOLL-INTERLEUKIN RECEPTOR HOMOLOGY-NUCLEOTIDE BINDING SITE-LEUCINE RICH REPEAT (*TIR-NBS-LRR*)-type receptor family proteins appear to be enriched in this transcript subset, and these enzymes were recently described as NAD cleaving enzymes that promote cell death [43]. Additionally, polygalacturonases, transporters, and calcium-binding proteins are also significantly enriched in SIV-grown pollen tubes, all of which are affiliated with successful pollen tube elongation. Thirty-three of the most significantly altered genes that appeared critical for pollen function in the pistil were investigated by reverse genetic analysis, resulting in the identification of two genes, *NUCLEOREDOXIN1* (*NRX1*) and *XIPOTL*, that significantly impact pollen tube guidance through the pistil.

*NRX1* encodes for a protein containing thioredoxin and C1-like domains [39]. As C1 domains often bind diacylglycerol, this domain architecture might implicate *NRX1* in lipid signaling. However, NRX1 is a cytoplasmic and nuclear-localized protein that possesses disulfide bond reductase activity. Therefore, this protein may be less likely to play a role in plant lipid metabolism and more likely to contribute to protein folding of critical pollen tube targets, based on the observation that *nrx1* mutants display male-specific segregation distortion [44]. *XIPOTL* encodes for one of three Arabidopsis *S*-adenosyl-*L*-methionine:phosphoethanolamine *N*-methyltransferases (PEAMT), which are required for phosphatidylcholine synthesis. Phosphatidylcholine is a major membrane phospholipid and precursor of phosphatidic acid, an important lipid signaling molecule [45]. Mutants of *NRX1* and *XIPOTL* display abnormal growth patterns compared to wild-type pollen tubes, such as approaching but not entering the ovule micropyle, growing toward the chalazal end instead of the micropylar end of the ovule, and multiple pollen tubes being attracted to the same ovule [39]. Overall, these observations suggest that *NRX1* and *XIPOTL* are necessary for accurate ovule targeting, but the mechanistic basis for these observations remains unresolved.

Another elegant approach to elucidate pollen tube factors that contribute to enhanced growth within the pistil made use of high-throughput RNA-sequencing and automated SNP detection in fertilization assays between multiple ecotypes. This approach allowed for delineating gene expression changes in crosses between Cape Verde Island-0 (Cvi) and Columbia (Col-0) ecotype reproductive tissues. Upon evaluation of the differentially expressed genes using RNA-seq analysis, 277 genes were determined to be expressed specifically from the pollen tube and 6838 genes were expressed specifically from the pistil tissue [40]. This high pistil-specific ratio is at least partially due to the excess of pistil tissue in the sample being analyzed, but still demonstrates that different gene expression patterns can be evaluated by identifying paternal and maternal genes by ecotype SNPs. While many of the pistil expressed genes remain to be evaluated, these results may indicate that numerous pistil-specific transcripts cooperate to facilitate pollen tube penetration through the pistil.

Despite these high-quality transcriptome studies, very few mutants have been isolated that are specifically compromised in their ability to penetrate the stigma-style interface. While there must be a continuum between mutants that impact growth in the style versus growth in the transmitting tract, we will define the former class of mutants as having specific impacts in the stigma-style interface as evidenced by reduced pollen tube penetration in SIV experiments. The Arabidopsis *O-FUCOSYLTRANSFERASE1* (*AtOFT1*) gene confers pollen tubes the ability to penetrate the stigma-style interface [46]. *AtOFT1* transcripts are expressed in pollen tubes and increase in abundance upon penetration through the stigma and style. *oft1* mutant pollen tubes were indistinguishable from wild-type when grown in vitro, but *oft1* mutant pollen tubes penetrated the stigma-style interface much slower compared to Col-0 pollen tube controls, resulting in an approximate 2000-fold decrease in *oft1* pollen transmission and a nearly 10-fold reduction in seed set. The stigma-style interface proved to be a significant barrier for *oft1*, as excision of the stigma-style interface [47] from the pistil allowed *oft1* mutant pollen to fertilize ovules at a much higher frequency and produce progeny [46].

The Arabidopsis genome contains 39 putative *OFT*-like genes that all bear amino acid sequence similarity to metazoan Protein *O*-FucosylTransferases (POFTs). POFTs utilize GDP-fucose as a sugar nucleotide donor to transfer *L*-fucose onto serine and threonine residues of target proteins which typically contain Epidermal Growth Factor (EGF) or Thrombospondin Repeat (TSR) domains in metazoan systems, and these post-translational fucosylation events often confer critical regulatory functions. For example, in the Notch signaling cascade, mono-*O*-fucosylation of the Notch extracellular domain potentiates interactions with cognate Notch ligands, such as Serrate, Delta, and Jagged [48,49,50,51,52]. These *O*-fucosylation events ultimately communicate mechanical information between cells to regulate cell adhesion and cell fate [53]. Substrates of *AtOFT1* remain to be elucidated. However, it is proposed that *oft1* mutant pollen tubes might fail to penetrate the stigma-style interface due to their inability to degrade the pistil cell wall extracellular matrix or that AtOFT1 might post-translationally modify other proteins that serve as receptors for receiving positional guidance cues from the stigma, style, and transmitting tract [54]. It is also important to note that several genes annotated as putative POFTs have recently been ascribed the unrelated function of serving as Rhamnogalacturonan-Rhamnosyl Transferases (RRTs), suggesting that this class of proteins could be participating in pollen tube RG-I deposition [55,56].

The endocytic pathway and players of vesicle-mediated transport have also been implicated in pollen tube penetration through the stigma [57]. The Arabidopsis *VACUOLAR PROTEIN SORTING41* (*VPS41*) gene encodes a novel factor that facilitates control of pollen-stigma interaction. *vps41* loss-of-function mutants display a phenotype of disrupted stigma/style penetration, resulting in male gametophyte sterility. VPS41 localizes to pollen tube late endosomes and tonoplasts and interacts with RAB GTPases (Figure 2A). The *vps41* mutants also display a disruption during the late stage of the endocytic pathway in pollen tubes, and disruption of VPS41 localization to the tonoplast compromises its function in elongating pollen tubes. These observations implicate the endocytic pathway in playing a crucial role in support of male gametophyte fertility, as vacuole biogenesis is proposed to affect turgor pressure regulation during pollen tube growth. It is also plausible that VPS41 facilitates endocytosis and degradation of plasma membrane-localized pollen tube receptors, such as the ANX-BUPS-LLG complex discussed below.

Additional work highlights the importance of regulated pectin metabolism in pollen tube stigma/style penetration. *VANGUARD1* is a pectin methyl esterase (PME) that is responsible for approximately 80% of the PME activity in pollen tubes. The *vgd1* mutants produce pollen tubes that successfully penetrate through stigmatic papillae cells but fail to rapidly elongate at the stigma-style interface [58]. Because this protein was secreted into the pollen tube cell wall, it was proposed that VGD1 either contributes to the structural dynamics of homogalacturonan de-esterification to rigidify pollen tube cell walls or potentially is secreted into the female tissue where it would facilitate pectin de-methylesterification followed by homogalacturonan degradation. In the latter model, VGD1 may serve to soften and passivate female tissues to facilitate rapid pollen tube expansion in the pistil, but this hypothesis remains to be investigated.

## 6. Growth in the Transmitting Tract

After penetrating the stigma-style interface, the pollen tube continues growing through the transmitting tract (TT). It is important to note that the pollen tube must balance two important functions during this time. First, the pollen tube must elongate over a substantial distance and successfully navigate toward an unfertilized ovule. Second, the pollen tube is literally built to burst, but it must not burst prematurely. Thus, positional guidance cues in the transmitting tract must encourage the pollen tube to elongate toward ovules and actively prevent premature pollen tube rupture.

Several recent studies have elegantly highlighted the complex mechanical signaling mechanisms that prevent premature pollen tube bursting in the transmitting tract. The *ANXUR* (*ANX*) *1* and *2* are pollen expressed *Catharanthus roseus* receptor-like kinase (CrRLK)-type proteins that coordinate proper rupturing time and release of the two sperm nuclei into the ovule. Interestingly, *anx1* or *anx2* mutant pollen tubes display premature rupturing in vitro. Similarly, double *anx1*/*anx2* mutant pollen tubes prematurely rupture in the transmitting tract or style tissue when applied to pistils [59]. As pollen tubes typically do not rupture until arriving at the egg apparatus in the ovary for fertilization, this phenotype suggests that these RLKs are involved in regulating pollen tube rupture.

Phenotypes that are similar to *anx1*/*anx2* mutants were observed in mutants of *BUDDHA’S PAPER SEAL* (*BUPS*) 1 and 2, a second pair of CrRLK-type receptor-like kinases [60,61]. Additionally, mutations in *RAPID ALKALINIZATION FACTORS* (*RALF*) *4* and *19* [60], as well as *LORELEI-LIKE GENE* (*LLG*) *2* and *3* [62,63], recapitulate *anx1*/*anx2* phenotypes. These initial observations led multiple groups to demonstrate that LLG, ANX, and BUPS proteins form a multi-protein receptor complex that perceives RALF4 and 19 peptides that are secreted from the pollen tube and actively prevent the pollen tube from bursting. RALF peptides are also secreted by ovules, and the ovule-secreted RALF34 is capable of displacing RALF4 or 19 from the ANX-BUPS-LLG receptor complex in vitro, leading to rapid and quantitative pollen tube bursting [60]. These observations suggest that RALF4 and 19 peptides are secreted from the pollen tube as it migrates through the transmitting tract to maintain pollen tube integrity. At the ovule, it is likely that peptides such as RALF34 displace these autocrine RALF peptides and facilitate pollen tube bursting at the correct location (i.e., in the synergid cell). Additionally, it should be noted that other members of the CrRLK family are known to phosphorylate and inactivate proton ATPases in response to RALF peptides [64], suggesting that RALF34-like peptides secreted from the ovule could potentially activate this pathway leading to pollen tube bursting.

Despite these advances, many questions remain about the functions that are performed by this complex signaling system (Figure 2B). For example, the downstream signaling outputs of the ANX-BUPS-LLG signaling complex in response to either RALF4/19 or RALF34-like peptides are unclear, and it is not precisely elucidated how this signaling complex dynamically controls pollen tube integrity. Intriguingly, BUPS RLKs physically interact with RopGEFs, which are known to control tip-localized growth behavior [61]. ROP1 regulates pollen tube exocytic traffic at the growing tip [65], and this protein is in turn activated by RopGEFs. A recent study found that *bups* mutant pollen tubes could be compressed while growing through small microfluidic channels similar to TipChip described above, but that *bups* pollen tubes ruptured shortly after exiting the narrow channel [66]. This observation suggests that the pollen tube undergoes significant mechanical stress when pressure at the growing tip is relieved. A constitutively activated ROP1 could partially alleviate many of the phenotypic defects associated with the *bups* mutation, suggesting that ROP1-mediated exocytosis is likely important for the mechanotransduction associated with pollen tube exit from the stigma-style interface, and that this process may be regulated by physical interactions between the BUPS intracellular domain, interacting RopGEF factors, and ROP1.

In addition to maintaining structural integrity, pollen tubes must follow long-range positional guidance cues in the transmitting tract (Figure 2C). In *Nicotiana tabacum*, arabinogalactan proteins (AGPs) are a predominant class of extracellular matrix proteins found in the stylar transmitting tract [67]. AGPs are hydroxyproline-rich glycoproteins that contain a high carbohydrate content and exhibit adhesiveness and cell surface localization. Due to their abundance in pistil tissues, AGPs have been implicated in pollen tube germination and elongation. A family of tobacco AGPs called Transmitting Tissue Specific (TTS) proteins directly promote pollen tube elongation in vivo and in vitro, and serve as pollen tube attractants [68]. TTS proteins adhere to the pollen tube surface and integrate into the pollen tube wall matrix. TTS glycans are hydrolyzed by pollen tube deglycosylating enzymes, and this process is regulated at the transcriptional, post-transcriptional, and post-translational levels to ensure that highly glycosylated TTS proteins accumulate along the transmitting tract. The function of TTS proteins in tobacco has been evaluated using loss-of-function mutants to demonstrate that highly reduced levels of TTS proteins correlate with reduced pollen tube growth rates. This correlation is also observed in Arabidopsis *agp6*; *agp11* double mutants, suggesting a conserved role of AGPs in pollen tube growth and elongation [69].

Genetic evidence also suggests a role for γ-amino-butyric acid (GABA) in pollen tube navigation through the transmitting tract. The Arabidopsis *POLLEN–PISTIL INCOMPATIBILITY* 2 (*POP2*) mutant is compromised in the degradation of GABA due to a mutation in GABA transaminase, which leads to 20-fold higher levels of GABA in the transmitting tract [70]. GABA stimulates pollen tube growth in vitro at low concentrations but is inhibitory at the concentrations experienced in the *pop2* mutant. GABA is likely perceived by calcium-permeable ion channels at the pollen tube tip that stimulate periodic calcium influx that is associated with pollen tube growth [71]. Under normal conditions, GABA forms a concentration gradient that increases along the transmitting tract, but this gradient is disrupted in the *pop2* mutant, which leads to reduced growth of pollen tubes through the pistil and female sterility. These observations suggest that the GABA gradient established in the pistil may serve as a long-range positional guidance cue to help pollen tubes navigate through this structure.

As pollen tubes navigate the transmitting tract, they must also be directed to unfertilized ovules by positional guidance cues from the ovule. The recently identified LURE peptides play a large role in this portion of pollen–pistil interactions. LUREs are small defensin-like peptides secreted from synergid cells that attract pollen tubes alone in vitro or in the context of the ovule [72,73]. LUREs bind to the pollen tube via tip-localized receptor-like kinase PRK6 [74]. Recently, the AMOR glycan (4-Me-glucuronosyl β(1→6) galactose), a potential degradation product of AGP glycans, was demonstrated to potentiate this signaling event, suggesting that multiple ovule-derived cues provide positional guidance for pollen tube navigation to the ovule [75].

## 7. Synergid Cell Penetration and Sperm Cell Discharge

**Figure 3:**
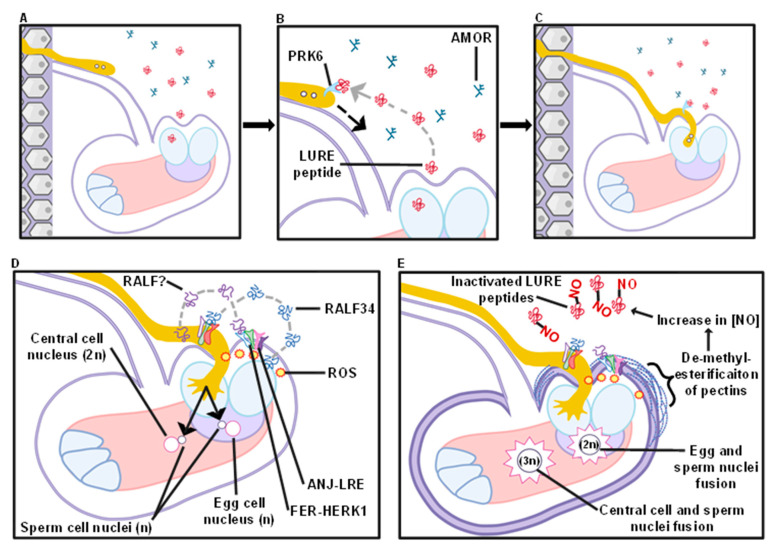
Schematic of a pollen tube being attracted to an ovule and synergid penetration. (**A**) The pollen tube exits the transmitting tract and moves along the funiculus. (**B**) While approaching the ovule, PRK6, a receptor-like kinase, binds LURE peptides secreted by the synergid cells, acting as a positive reinforcement signal. AMOR glycans, potentially attached to AGPs, potentiate this attractive signal. (**C**) The pollen tube enters the ovule by penetrating a synergid cell. (**D**) As the pollen tube penetrates the filiform apparatus, and subsequently the synergid, it is prompted to burst. The pollen tube’s ANX1/2-BUPS1/2-LLG2/3 signaling complex binds RALF34, secreted by the filiform apparatus, and the ovule’s FER-HERK1/ANJ-LRE signaling complex potentially binds unidentified RALFs, secreted by the pollen tube. FER-HERK1/ANJ-LRE signaling results in Reactive Oxygen Species (ROS) accumulation in the filiform apparatus, facilitating pollen tube bursting. (**E**) Sperm cell fuses with the egg and central cells to form the new embryo and endosperm tissue, respectively. Pectins surrounding the ovule become de-methylesterified. Nitric oxide (NO) species are released into the surrounding area. NO species nitrosate and inactivate LURE peptides, working synergistically with the rigidifying pectin to prevent polytubey.

At an unfertilized ovule, the pollen tube must penetrate the synergid cells, rupture, and release sperm cells, which represents yet another unique cell penetration event (Figure 1D and Figure 3). The fertilized ovule must also signal to other pollen tubes that the fertilization event has been completed to avoid polytubey, or the invasion of an ovule by multiple pollen tubes. Genetic analysis indicates that these processes are primarily controlled by the CrRLK-type receptor-like kinases FERONIA (FER), HERCULES1 (HERK1), and ANJEA (ANJ). Similar to the ANX-BUPS-LLG signaling complex described previously, FER, HERK1, and ANJ associate into a larger signaling complex with the LORELEI (LRE) co-receptor.

The *FERONIA*/*SIRENE* (*FER*/*SRN*) receptor-like kinase is expressed in synergid cells and localizes to the filiform apparatus to control pollen tube behavior during synergid cell penetration [76]. Null *fer* mutants displayed female semi-sterility, pollen tube coiling within the synergid, and frequent polytubey [76]. Thus, *FER* confers female gametophytes the ability to control pollen tube behavior following synergid cell penetration. The establishment of *FER*’s role in pollen tube signaling has allowed for subsequent elucidation of other effector proteins and members in its signaling pathway.

Mutations in the GPI-anchored protein LRE were isolated independently but displayed all of the hallmarks of the *fer* mutant, including female sterility, polytubey, and pollen tube coiling in the synergid cell without pollen tube bursting [77]. Similar phenotypes were also recently described for double *herk1*/*anj* mutants [78], indicating that FER, LRE, HERK1, and ANJ all participate in the same pathway. Further analyses indicate that FER, LRE, HERK1, and ANJ interact with each other in both heterologous systems as well as in planta, and that all of these proteins localize to the filiform apparatus, which is the site of pollen tube entry into the synergid [78]. These observations indicate that a FER-HERK1/ANJ-LRE receptor complex exists in the filiform apparatus, and this signaling complex plays a major role in facilitating pollen tube rupture at the synergid as well as blocking polytubey after fertilization is complete.

The molecules that activate the FER-HERK1/ANJ-LRE complex and the mechanisms of downstream signaling leading to pollen tube rupture and blocking additional pollen tubes are less clear (Figure 3D). It is very likely, based on other CrRLK signaling systems, that the FER-HERK1/ANJ-LRE signaling complex binds to RALF peptides to initiate signal transduction, but the precise RALF isoform ligand has not been elucidated. In other signaling contexts, FER can bind RALF1 [64] and the ovule-secreted RALF34 peptide can cause pollen tube bursting [60], so these RALF peptides may represent reasonable candidate ligands. Several outputs of the FER-HERK1/ANJ-LRE signaling complex have been revealed and likely impact the process of pollen tube rupture. First, the seven transmembrane domain MLO-related protein NORTIA (NRT) is re-localized in the filiform apparatus in a FER- and pollen tube-dependent manner, although the function of NRT and thus the mechanistic outcome of this re-localization event is unclear [79]. Second, the FER-HERK1/ANJ-LRE signaling complex causes the formation of reactive oxygen species (ROS) with a maximum concentration at the filiform apparatus. Intriguingly, multiple lines of evidence suggest that ROS accumulation can lead to pollen tube rupture, and that depletion of ROS at the filiform apparatus recapitulates phenotypes associated with FER-HERK1/ANJ-LRE dysfunction, including pollen tube coiling and polytubey [80]. Finally, FER, and likely its additional signaling complex components, elicits a series of responses to prevent an ovule from being entered by another pollen tube (Figure 3E). FER seems to control or respond to the increased presence of de-methylesterified pectin at the filiform apparatus, and inhibition of pectin de-methylesterification causes increased polytubey. Additionally, the presence of de-methylesterified pectin causes an increase in nitric oxide species in a FER-dependent manner, which leads to the protein nitrosation and inactivation of LURE peptides [81]. Thus, the FER-HERK1/ANJ-LRE signaling complex participates in multiple signaling processes that lead to pollen tube rupture, establishing a blockade to prevent additional pollen tubes from entering the fertilized ovule, and reducing efforts to attract additional pollen tubes.

The pollen tube also likely contributes to correctly-timed bursting within the synergid cell. Upon investigating which transcription factors display elevated expression during pollen development and pollen tube growth, *MYB97*, *101*, and *120* formed a closely related pollen tube-expressed subclade [82], leading to the hypothesis that MYBs may regulate pollen tube gene expression in response to growth through the pistil. Transcriptome microarray analysis was used to identify differentially expressed genes between wild-type and *myb97*/*101*/*120* triple mutants, revealing that only 48 genes were differentially expressed by these transcription factors, encompassing enriched functional categories of transporters, carbohydrate-active enzymes, and small secreted proteins. One of the small, secreted proteins identified is a cysteine-rich thionin that is specifically expressed in pollen tubes and requires the activity of all three MYB transcription factors for expression. As thionins are recognized for their ability to form transmembrane pores, it is proposed that this function might also aid in conferring the pollen tube its ability to burst in a controlled manner.

## 8. Sperm Cell Fusion

The final physical barriers of double fertilization require that released sperm cells fuse separately with the egg and central cell to form the new embryo and endosperm tissue, respectively. Careful cytological analysis using sperm cell-localized photo-convertible fluorescent proteins suggest that sperm cells are equivalent and can fuse with either nuclei [83] and that both fusion events are accomplished in less than 10 min. The molecular players that facilitate this process are coming into view. HAPLESS2 (GENERATIVE CELL SPECIFIC 1) (HAP2/GCS1) is a membrane protein that displays sequence similarity to viral and animal membrane fusion domains. HAP2/GCS1 is expressed in sperm cells and undergoes regulated trafficking to the sperm cell plasma membrane in response to EGG CELL 1 (EC1) peptides that are secreted from the egg [84]. HAP2/GCS1 translocation to the sperm cell plasma membrane facilitates cell–cell interactions between sperm and egg as well as the ultimate sperm-egg fusion event, suggesting that this protein performs the necessary events that mediate cellular fusion.

GAMETE EXPRESSED 2 (GEX2) is a second plasma membrane-localized protein of unknown function that participates in cellular interactions between sperm and egg cells. The *gex2* mutants exhibited male sterility that was ultimately linked to failed sperm fusion events, and, in an elegant assay, the authors of this study demonstrated that *gex2* mutant sperm cells adhere to protoplasted egg cells less frequently than wild-type sperm [85]. Interestingly, *hap*/*gcs1* mutants were not compromised in egg-sperm cell interactions, suggesting that *GEX2* plays an important role in this cellular interaction.

Recent work has shed light on how sperm cells select a fusion target. A small family of *DOMAIN OF UNKNOWN FUNCTION 679 Membrane Protein 9* (*DMP*) genes in Arabidopsis were found to be transcriptionally co-expressed with *GEX2* and encode protein products that were likely expressed in sperm cells. Double *dmp8*/*dmp9* mutants show various hallmarks of reduced male fertility, including reduced seed set and reduced pollen transmission, but these mutants also display the curious phenotype that they often produce aborted seeds with fertilized central cells, but unfertilized embryos [86]. Further investigation revealed that DMP8/9 are localized to the sperm cell membrane and that *dmp8*/*dmp9* mutants were more often compromised in egg-sperm cell fusion events than in central cell-sperm fusions. These observations suggest that DMP proteins may contribute to cell fusion selectivity and that future work may reveal a role in other unique DMP family members toward mediating central cell-sperm fusion events.

The information presented above suggests a model describing the unique functions of these proteins in overcoming the gamete fusion physical barrier. GEX2 seems to play a primary role in tethering cells together to facilitate membrane fusion, while HAP2/GCS1 mediates the formal process of membrane fusion. The recently identified DMP proteins may contribute to selectivity of the cell fusion events, and EC1 serves as a secreted signal that prepares the sperm cells for fusion prior to arriving at the egg or central cell. Despite this progress, it remains unclear whether proteins or other signals displayed on the egg or central cell have analogous or complementary activities that mediate cellular tethering, fusion, or cell fusion selectivity.

## 9. Conclusions and Future Perspectives

Pollen tube penetration through the various cell types of the pistil requires the coordinated signaling of growth factors that facilitate pollen tube elongation and navigation [70,72,75] as well as the secretion of hydrolytic enzymes to degrade portions of pistil cells [58]. While the genetic requirements for this process are beginning to come into view, aspects of the molecular mechanisms underlying pollen tube penetration remain unclear. Below, we suggest several future avenues worthy of investigation.

It is quite fascinating that pollen tubes are capable of penetrating through extracellular spaces that are only a few hundred nanometers wide in vivo but lack this capacity in controlled in vitro settings. It is certainly clear that pollen tubes secrete an array of cell wall modifying enzymes that can degrade and capacitate cell wall material along the female reproductive tract [9]. However, this situation leads to an array of follow-up questions. How is it possible that the cell wall-degrading enzymes that facilitate pollen tube penetration through the female tissue do not also degrade the pollen tube cell wall? Numerous observations described above indicate that genetic disruption of pollen tube cell wall biosynthesis cause severe impacts on pollen tube elongation, so how is it possible that cell wall degrading enzymes targeted at female tissues do not have similar effects? It is interesting in this regard to note that (1) many of the cell wall-degrading/modifying enzymes secreted by the pollen tube are uniquely expressed in this cell type, and (2) that the precise substrate specificity of major classes of wall degrading enzymes, such as PMEs, polygalacturonases, and pectate lyases is simply not clear due to the lack of availability in defined substrates [87]. Therefore, it is possible that pistil pectin chemistry or unique aspects of cell wall polymers may passivate this structure to specific deconstruction by pollen tube-specific cell wall degrading enzymes. It is also important to consider that pollen tube mechanical signaling may contribute to the penetration process by activating certain pistil tissues to secrete their own hydrolytic enzymes to “autodegrade” their cells and passivate the female tissue for pollen tube penetration.

We have illustrated a wide array of signaling cascades and feedback mechanisms that pollen tubes utilize in order to successfully locate and fertilize an ovule. Recent work indicates that the BUPS-ANX-LLG receptor complex plays a critical and dynamic role during pollen–pistil interactions by maintaining pollen tube mechanical stability in the stigma/style interface and transmitting tract, while also facilitating bursting at the synergid. During mechanical signaling at the stigma-style interface, it is clear that ROP1-mediated polarized secretion participates in this process [66], but it will be important to identify the cargoes that are delivered by this system to the pollen tube tip to facilitate increased mechanical strength. Additionally, it will be important to dissect the downstream signaling components that allow pollen tubes to rupture in the presence of ovule-derived RALF peptides, such as RALF34. The phenotype of *oft1* and *vps41* mutants are similar (though not identical) to many of the mutants that are impacted in this pathway, and their roles in regulating BUPS-ANX-LLG signaling should be investigated in greater detail. *O*-fucosylation events or other post-translational modifications might confer receptors or other proteins the ability to mechanically interact with their respective pistillar receptors to signal that navigation and penetration are taking place. This possible mechanical interaction could result in female tissue reorganizing or further cooperation between the pollen tube and the pistil.

Although a large body of recent work has highlighted mechanisms by which pollen tubes are attracted to ovules, growth attractants in the transmitting tract remain less clear. It will also be important to understand whether other factors secreted into the transmitting tract have long range guidance functions or serve to passivate the transmitting tract for easier pollen tube growth. Transcriptomic studies have attempted to delineate differences between pollen tube and pistillar cell transcriptomes, however, a large number of pistillar transcripts remain to be investigated. Thus, we also ask the questions: Are the pistillar cells receiving a signal from the pollen tube and facilitating its navigation? Or does this navigation depend on the pollen tube’s ability to navigate and penetrate independently of female tissue aid?

As noted above, many questions remain unanswered regarding mechanical signaling at physical barriers during pollen–pistil interactions, with many of these mechanistic understanding gaps at the stigma-style interface or during growth in the transmitting tract. Perhaps these barriers could be better mechanistically defined by taking advantage of recent technological advances, which we highlight here. First, two-photon microscopy can provide a viable experimental platform to observe the behavior of live pollen tubes growing through pistil tissue [27]. Thus, this approach may prove useful in the characterization of mutants that are impaired in penetration events within the pistil. Second, a growing palette of mechanotransduction reporters have been developed in recent years that allow for direct measurement of cellular forces. For example, modular FRET-based tension sensors have been reported that are capable of measuring piconewton forces via fluorescent outputs [88]. These sensors can be modified with interaction-specific recognition molecules, facilitating the measurement of discrete forces in a cell-type specific manner, and thus they may be useful for measuring penetrative force dynamics during pollen tube invasion. Lastly, a large suite of techniques have been employed to mark and profile specific cell types that functionally interact during cell migration events in non-plant systems [89]. For example, proximity labeling and fluorescent protein reconstitution methods may hold some promise for specifically investigating the interface between the pollen tube and the invaded pistil tissue.

Investigating mechanical signaling in plant systems is quite difficult because the majority of cells in plant tissues adhere to one another though cell wall matrices. Pollen–pistil interactions represent a unique example of plant cell–cell interactions and cell–cell signaling that can be precisely controlled by the simple addition of pollen. While this system has vastly enhanced our understanding of mechanical signaling in the pistil and beyond, many questions remain to be resolved. Utilization of the approaches outlined above, as well as addressing some of these critical questions, will likely have important impacts on our understanding of pollen–pistil interactions, as well as broader questions associated with how plant cells interact to form the plant body.
